# Nuclear genes involved in mitochondrial diseases caused by instability of mitochondrial DNA

**DOI:** 10.1007/s13353-017-0424-3

**Published:** 2018-01-17

**Authors:** Joanna Rusecka, Magdalena Kaliszewska, Ewa Bartnik, Katarzyna Tońska

**Affiliations:** 1 Institute of Genetics and Biotechnology, Faculty of Biology, University of Warsaw, Pawinskiego 5a, 02-106 Warsaw, Poland; 2 Institute of Biochemistry and Biophysics, Polish Academy of Sciences, Pawinskiego 5a, 02-106 Warsaw, Poland

**Keywords:** Mitochondrial diseases, Nuclear genes, Mitochondrial DNA instability, Mitochondrial DNA deletions, Depletion

## Abstract

Mitochondrial diseases are defined by a respiratory chain dysfunction and in most of the cases manifest as multisystem disorders with predominant expression in muscles and nerves and may be caused by mutations in mitochondrial (mtDNA) or nuclear (nDNA) genomes. Most of the proteins involved in respiratory chain function are nuclear encoded, although 13 subunits of respiratory chain complexes (together with 2 rRNAs and 22 tRNAs necessary for their translation) encoded by mtDNA are essential for cell function. nDNA encodes not only respiratory chain subunits but also all the proteins responsible for mtDNA maintenance, especially those involved in replication, as well as other proteins necessary for the transcription and copy number control of this multicopy genome. Mutations in these genes can cause secondary instability of the mitochondrial genome in the form of depletion (decreased number of mtDNA molecules in the cell), vast multiple deletions or accumulation of point mutations which in turn leads to mitochondrial diseases inherited in a Mendelian fashion. The list of genes involved in mitochondrial DNA maintenance is long, and still incomplete.

## The mitochondrion and its genome

Mitochondria are cytoplasmic organelles with a double phospholipid membrane and are present in almost all eukaryotic cells. Mitochondria are necessary for cell form and function. Their best recognized role is to generate energy by oxidative phosphorylation, but they also play a key role in synthesis of iron–sulfur centers, fatty acid oxidation, chemical signaling (Ca^2+^ signaling) and programmed cell death. The number of mitochondria in the cell varies and depends on the cell type and energy requirement, where cells with greater energy needs have more mitochondria than cells with smaller needs (Hudson and Chinnery 2006). Mitochondria are considered to be semi-autonomous, because in the course of evolution they have maintained their own small genome, known as mitochondrial DNA (mtDNA). The mitochondrial genome was discovered by Nass and Nass in 1963 (Nass and Nass 1963). In the same year, Schatz isolated mtDNA from *Saccharomyces cerevisiae* (Schatz 1963; Holt and Reyes 2012). mtDNA is required for production of key catalytic subunits of the mitochondrial respiratory chain complexes and therefore is essential for oxidative ATP production. In humans, it is a circular molecule of 16.5 kb carrying only 37 canonical genes. The mtDNA genes encode: 2 rRNAs, 22 tRNAs and 13 of 83 genes for respiratory chain subunits (*MT-ATP6, MT-ATP8, MT-CO1, MT-CO2, MT-CO3, MT-CYB, MT-ND1, MT-ND2, MT-ND3, MT-ND4, MT-ND4L, MT-ND5, MT-ND6*). Additionally, according to the newest discoveries, human mtDNA also encodes three short peptides, humanin, *gau* proteins and MOTS-c, with important biological functions, e.g., humanin plays a significant role in protecting neurons from apoptosis in Alzheimer’s disease (Shokolenko and Alexeyev 2015; Capt et al. 2016).

All other proteins (over 2000) required for the proper function of all mitochondrial biochemical pathways, including the rest of the subunits of respiratory complexes, are encoded by nuclear genes. This means that mitochondrial DNA expression, maintenance, copy number regulation, and repair processes rely on the nuclear genome (Tyynismaa et al. 2005; Capps et al. 2003; DeBalsi et al. 2016; Scheibye-Knudsen et al. 2015).

Several features make mitochondrial DNA unique, for example in mammals it is maternally inherited (Chen et al. 2010). Moreover, there are up to thousands of mtDNA copies in each cell (Suomalainen and Isohanni 2010). When all the mtDNA molecules have the same sequence (wild or mutated) it is called homoplasmy while heteroplasmy implies the mixture of two or more types of mtDNA (for example wild type and mutant). The heteroplasmy level of pathogenic variants correlates with the phenotype to some extent.

## Genetics of mitochondrial diseases

Mitochondrial diseases are defined by a respiratory chain dysfunction and in most of the cases manifest as multisystem and multiorgan disorders with predominant expression in muscles and nerves. Generally, the prevalence of mitochondrial disease is around 1:10,000 and is similar to diseases like phenylketonuria or spinal muscular atrophy but the exact frequencies vary between different populations and are not known for many of them. Prevalence of mitochondrial diseases is different in children (6.2:100,000) and adult patients (1:4300 affected or at risk) (Lightowlers et al. 2015). Moreover, the prevalence varies between populations of patients, e.g., prevalence of mitochondrial diseases in Spanish adult population (older than 14 years) is 5.7:100,000 (Arpa et al. 2003), in Australia 4.7:100,000 (Skladal et al. 2003).

Gorman et al. (2015) showed that mitochondrial disease is caused by mutations in nuclear genes in 2.9 per 100,000 adults in North East England.

Diseases caused by mtDNA mutations are maternally inherited, while those caused by mutations in nuclear genes encoding proteins more or less directly engaged in the function of the oxidative phosphorylation system (OXPHOS) are inherited in a Mendelian fashion (Wortmann et al. 2015). An interesting subgroup of mitochondrial disorders results from large deletions of mtDNA or its depletion. While single large mtDNA deletions occur spontaneously and are in most cases not transmitted from a mother to her children, multiple mtDNA deletions and depletion have Mendelian inheritance (Wong 2013; Dinwiddie et al. 2013; Lightowlers et al. 2015). The former is the result of the fact that the maintenance of mtDNA relies on proteins encoded in the nuclear genome.

Mitochondrial disorders associated with disturbed mtDNA stability (copy number and quality) are collectively called mitochondrial maintenance diseases or mtDNA depletion syndromes. The main feature of those disorders is rearrangement of the mitochondrial genome seen as multiple deletions of mitochondrial DNA molecules (the presence of multiple classes of mtDNA molecules of different lengths) and/or decrease of mtDNA copy number in cells, known as mtDNA depletion (Krishnan et al. 2008; Nicholls et al. 2014; Wong 2013; Gorman et al. 2015).

## Mitochondrial DNA maintenance

Although mitochondrial DNA is not wound onto histone structures, it does not freely float in mitochondrial matrix. It is covered mainly by TFAM protein discovered as a transcription factor, but mainly engaged in forming the proper shape of the mitochondrial nucleoid and in copy number control (details later). The number of mtDNA molecules in one nucleoid is still being discussed — it seems to be one to a few. Obviously, replication plays the main role in the maintenance of mitochondrial DNA (Campbell et al. 2012).

## mtDNA replication machinery

Initially, the strand displacement model (SDM) of replication was suggested but it was partially incorrect due to artifacts which occurred during the preparation process. An updated version of the SDM is called the RNA intermediate throughout the lagging-strand (RITOLS) model. Both models imply the presence of two origins of replication (ori, O), one on the heavy (H) strand and one on the light strand (L), called OH and OL respectively (Nicholls et al. 2014; Holt and Reyes 2012). OH is located within the non-coding region (NCR) of mtDNA, whereas OL is at two-thirds of the mtDNA length, within a cluster of tRNA genes. Replication starts from OH; polymerase adds nucleotides to an RNA primer and synthesis of the light strand starts only after OL has been reached. The main distinction is that the displaced maternal heavy strand is supposed to be naked in SDM and covered by short RNA fragments in the RITOLS model, but the main assumption, asynchronous replication, is common for both of them (McKinney and Oliveira 2013).

In 2000, Holt and colleagues proposed a new, synchronous model of mitochondrial DNA replication called COSCOFA (conventional strand–coupled Okazaki fragment associated). This model implies that synthesis is initiated bidirectionally from multiple origins of replication at ori zone (ori z). The leading H strand is synthesized continuously and the lagging L strand is formed without delay as Okazaki fragments (Holt et al. 2000).

It is suspected that different types of mitochondrial replication systems are present in various tissues or depend on the energy state of mitochondria and cells (Martin-Garcia 2013).

The most important enzyme taking part in mtDNA replication is DNA polymerase gamma. Further proteins involved in this process are: Twinkle helicase, single-stranded DNA binding protein (mtSSB; may stabilize the displaced maternal H strand) (Holt and Reyes 2012), topoisomerase (introduces the breakpoint in mtDNA and separates strands), mitochondrial RNA polymerase (mtRNAP; provides RNA primers for initiation of replication), RNaseH1 and mitochondrial DNA ligase III (Young and Copeland 2016). Defects in the mitochondrial DNA replication process result in a single or multiple mutations in mtDNA and lead to multiple deletions and/or depletion of mtDNA molecules (Hudson et al. 2007).

## Genes encoding proteins involved in mitochondrial DNA replication

**Fig. 1:**
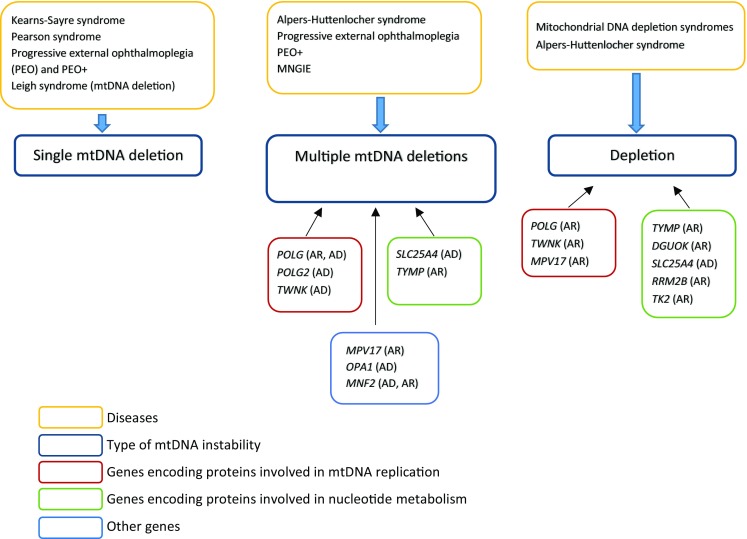
Mitochondrial DNA instability types with their molecular backgrounds and diseases they cause. AR – autosomal recessive, AD – autosomal dominant

The catalytic subunit of DNA polymerase gamma (encoded by the *POLG* gene) and its processivity factor (encoded by the *POLG2* gene) together with Twinkle helicase (encoded by the *TWNK* gene), DNA replication helicase/nuclease 2 (encoded by the *DNA2* gene), single-stranded DNA binding protein 1 (encoded by the *SSBP1* gene), primase and polymerase (DNA-Directed) (encoded by the *PRIMPOL* gene), and mitochondrial genome maintenance exonuclease 1 (encoded by the *MGME1* gene) play the key role in mitochondrial DNA maintenance and replication processes (Fig. 1).

### *POLG* and *POLG2* genes

One of the most important proteins encoded by the nuclear genome involved in replication, expression, maintenance, and repair of mitochondrial DNA is polymerase gamma (Polγ). Polγ is the only DNA polymerase involved in mtDNA replication present in the human mitochondrion (García-Gómez et al. 2013).

The holoenzyme is composed of a catalytic subunit POLG encoded by the *POLG* gene (15q26.1, 23,491 bp, 23 exons) and a homodimer of accessory subunits POLG2 encoded by the *POLG2* gene (17q23.3, 26,283 bp, 8 exons) (Johnson and Johnson 2001; Oliveira et al. 2015; Hudson and Chinnery 2006).

POLG has a catalytic core with 3′-5′ exonuclease activity responsible for proofreading (26–418 amino acids), a linker domain (419–755 amino acids), and 5′-3’polymerase activity responsible for replication (756–1239 amino acids) (Oliveira et al. 2015; Hudson and Chinnery 2006).

The subunit encoded by the *POLG* gene is necessary for proper function of the enzyme, it enhances enzyme activity by simultaneously accelerating the polymerization rate and suppressing exonuclease activity (Szymanski et al. 2015; Johnson and Johnson 2001; Lee et al. 2009). Polymerase gamma is considered a high fidelity polymerase introducing less than 2 × 10^−6^ errors per nucleotide (Hudson and Chinnery 2006). POLG2 increases the affinity for DNA molecules (DiRe et al. 2009; Szymanski et al. 2015).

Mutations affecting polymerase gamma result in a wide range of genetic syndromes with many mtDNA mutations, deletions, multiple deletions, and depletion of mitochondrial DNA (Linkowska et al. 2015; Hudson and Chinnery 2006). Diseases associated with Polγ dysfunction caused by mutations in the *POLG* gene include mitochondrial DNA depletion syndrome 4A (Alpers type, MIM 203700), a fatal infant disease with epilepsy and drug induced liver failure, mitochondrial DNA depletion syndrome 4B (MNGIE type, MIM 613662) with gastrointestinal involvement, mitochondrial recessive ataxia syndrome (includes SANDO and SCAE, MIM 607459), and relatively benign progressive external ophthalmoplegia autosomal dominant 1 (MIM 157640) and autosomal recessive 1 (MIM 258450) (Naïmi et al. 2006). All the above-mentioned diseases, which differ in severity and range of symptoms, may be caused by the same spectrum of *POLG* mutations. Progressive external ophthalmoplegia with mitochondrial DNA deletions, autosomal dominant 4 (MIM 610131) develops as a result of mutations in the *POLG2* gene.

### TWNK

Polymerase γ cooperates with TWINKLE helicase (also known as PEO1) encoded by the *TWNK* gene (10q24.31, 11,866 bp, 6 exons). TWINKLE is a mitochondrial 5′-3′ helicase necessary for replication of human mitochondrial DNA (Milenkovic et al. 2013; Tyynismaa et al. 2004). It binds to and unwinds double-stranded DNA (dsDNA) by breaking hydrogen bonds between annealed nucleotide bases and separating to single strands (Tyynismaa et al. 2005; Korhonen et al. 2003; García-Gómez et al. 2013; Cieskielski et al. 2016; Lamantea et al. 2002).

Mutations in the *TWNK* gene studied in cell cultures and deletor mice resulted in blocking of the replication process, accumulation of intermediates and finally in multiple mtDNA deletions (Goffart et al. 2009).

Known mutations result in insufficient mitochondrial DNA synthesis and lead to deletions and depletion of mtDNA (Nikkanen et al. 2016; Paramasivam et al. 2016). They are a frequent cause of progressive external ophthalmoplegia with mitochondrial DNA deletions, autosomal dominant 3 (MIM 609286) but in rare cases may lead to recessive diseases like mitochondrial DNA depletion syndrome 7 (hepatocerebral type) (MIM 271245) and Perrault syndrome 5 (MIM 616138),

### SSBP1

Stabilization of the replication fork through preventing binding strands of a replicated fragment of DNA from forming secondary structures and degradation is the main role of single stranded DNA binding protein 1 (SSBP1) encoded by the *SSBP1* gene (7q34, 12,180 bp, 9 exons). SSBP1 interacts with polymerase gamma and helicase Twinkle and strengthens their functions (Hudson and Chinnery 2006; Ruhanen et al. 2010).

Studies on *Saccharomyces cerevisiae RIMI* null mutants (*RIMI* encodes ssDNA binding protein) (Van Dyck et al. 1992) and mutants in the *lopo* (*low power*) gene from *Drosophila melanogaster* (which affect the mitochondrial single-stranded DNA-binding protein) (Maier et al. 2001) showed depletion of mitochondrial DNA and confirmed that this protein is necessary for replication and maintenance of mtDNA. In HeLa cell cultures with silenced *SSBP1* the mtDNA/nDNA ratio decreased and synthesis of the D-loop was affected (Ruhanen et al. 2010). No pathogenic variants in this gene have been described.

### PRIMPOL

The *PRIMPOL* gene (4q35.1, 52,347 bp, 16 exons) encodes nuclear and mitochondrial primase and DNA directed polymerase. PRIMPOL plays a key role in mtDNA replication initiation. Moreover, it enables the replication machinery to replicate past DNA lesions (translesion synthesis, TLS), e.g., in apurinic/apyrimidinic sites (AP sites). This protein is present both in the nucleus and mitochondria. Silencing of the *PRIMPOL* gene in human fibroblasts leads to multiple mtDNA deletions and depletion. A *PRIMPOL* mouse knockout is viable but mtDNA replication deficiency is observed on the cellular level. This confirms that absence of PRIMPOL has multiple adverse effects on mtDNA synthesis (García-Gómez et al. 2013). Mutations in this gene have been shown to result in autosomal dominant myopia (type 22, MIM 615420).

### DNA2

A member of the DNA2/NAM7 helicase family, DNA2 is a DNA replication helicase/nuclease 2 enzyme encoded by the *DNA2* gene (10q21.3, 58,458 bp, 22 exons). It has nuclease, helicase, and ATPase activity and interacts with polymerase gamma by stimulating its catalytic activity. DNA2 removes RNA primers and stabilizes mtDNA structure during the replication process; thus, playing an important role in the maintenance of mitochondrial DNA. Moreover, DNA2 participates in repair of small DNA lesions induced by oxidation, alkylation or spontaneous hydrolysis and is critical for long-patch base-excision repair (LP-BER) (Ronchi et al. 2013).

Mutations in *DNA2* are associated with recessive Seckel syndrome 8 (MIM 615807) and dominant progressive external ophthalmoplegia with mitochondrial DNA deletions (MIM 615156).

### MGME1

The *MGME1* gene (20p11.23, 22,529 bp, 8 exons) encodes mitochondrial genome maintenance exonuclease 1 (MGME1) protein probably involved in mtDNA repair (Uhler et al. 2016).

MGME1 removes flaps (last 20–50 nt) during replication and enables the processing of mtDNA ends due to the ability to cleave dsDNA in both 5′-3′ and 3′-5′ directions. Thus this exonuclease can cut 5′ and 3′ flaps. Moreover, MGME1 may enhance exonuclease activity of polymerase gamma (Nicholls et al. 2014; Uhler et al. 2016).

Studies on human fibroblast culture with null *MGME1* show that the absence of MGME1 leads to large mtDNA rearrangements such as deletions and depletion. Significant lengthening of the D-loop leading to incorrect processing of the mtDNA 5’end was observed as well (Nicholls et al. 2014).

*MGME1* loss-of-function mutations lead to mitochondrial disease with DNA depletion, deletions, duplications, and rearrangements and result in mitochondrial DNA depletion syndrome 11 (MIM 615084).

### RNASEH1

Ribonuclease H1, encoded by the *RNASEH1* gene (2p25.3, 33,559 bp, 14 exons) is an endonuclease involved in DNA replication and repair processes both in the nucleus and mitochondria, but in mitochondria it is the only ribonuclease of that type while in the nucleus there are two (besides ribonuclease H2). Ribonuclease H1 specifically digests double–stranded DNA-RNA hybrids and is necessary to produce primers for mtDNA replication.

It was shown, both in mice and human, that loss of RNaseH1 activity disturbs mtDNA replication. In mice, a knockout of *Rnaseh1* leads to embryonic lethality with mtDNA depletion (Cerritelli et al. 2003). In human, mutations in *RNASEH1* have been recently attributed to autosomal recessive PEO with mtDNA deletions (MIM 616479) (Reyes et al. 2015).

## Genes encoding proteins involved in transcription of mtDNA

The transcription machinery is not only essential for gene expression but also mtDNA copy number regulation as it is responsible for the synthesis of the primers for replication. Mitochondrial RNA polymerase POLMRT and a set of transcription factors: TFAM, TEFM, and TFB2M are responsible for that process.

The human mitochondrial genome contains two transcription promoters: LSP and heavy-strand promoter 1 (HSP1) and HSP2 (Lodeiro et al. 2012). Initiation of transcription from HSP promoter is only POLMRT dependent. POLMRT generates short RNA primers near oriL (during the replication process this region becomes single stranded and forms a loop structure). Efficient initiation of transcription from LSP requires cooperative action of POLMRT and transcription factors TFAM and TFB2M (Litonin et al. 2010).

### POLRMT

Mitochondrial RNA polymerase encoded by the *POLRMT* gene (19p13.3, 23,346 bp, 21 exons) is responsible for transcription of the mitochondrial genome and also provides primers for mtDNA replication, therefore all changes in the enzyme structure (or transcription complex) may impact mtDNA stability. POLRMT together with TFAM, TEFM, and TFB2M forms a transcription complex (Kühl et al. 2016; Posse et al. 2015; Minczuk et al. 2011).

TFB1M and TFB2M interact directly with POLRMT, help in promoter recognition, and increase transcription efficiency 100–200-fold as compared with RNA polymerase alone (Falkenberg et al. 2002; Litonin et al. 2010).

### TFAM

*TFAM* (10q21, 14,088 bp, 9 exons) is the mitochondrial transcription factor A coding gene also known as *TCF6L3* or *mtTFA*. TFAM is a key activator of mitochondrial transcription, plays an important role in mitochondrial DNA replication and copy number regulation, and is crucial for mitochondrial biogenesis. TFAM expression and turnover depends on the interaction between POLMRT, TFAM, and mitochondrial DNA (Picca and Lezza 2015; Kang et al. 2007; Ekstrand et al. 2004). In vitro experiments show that equimolar amounts of TFAM and mtDNA template result in the maximal transcription level (Litonin et al. 2010). Change in *TFAM* expression results in change of the protein level and influences mtDNA copy number (it is directly proportional). A mutation in the *TFAM* gene has been recently described as a cause of neonatal liver failure with mtDNA depletion (Stiles et al. 2016).

### TEFM

The mitochondrial transcription elongation factor encoded by the *TEFM* gene (17q11.2, 7933 bp, 4 exons) is responsible for transcript elongation. TEFM forms a complex with mitochondrial RNA polymerase, interacts with its catalytic domain, enhances processivity (Minczuk et al. 2011), and probably regulates the DNA replication initiation process (Posse et al. 2015). It was shown that when TEFM is absent in a mitochondrial transcription machinery model transcription is terminated and total transcript levels were significantly lower and transcripts were shorter. This indicates that TEFM is essential for full-length mtDNA transcript formation (Posse et al. 2015).

### TFB2M

Mitochondrial transcription factor B2, also known as mitochondrial 12S rRNA dimethylase 2 or mitochondrial dimethyladenosine transferase 2 mtTFB2, encoded by the *TFB2M* gene (1q44, 25,703 bp, 8 exons) is a part of the mtDNA transcription complex (Moustafa et al. 2015).

Mutations in *POLMRT*, *TEFM*, and *TFB2M* have not been described yet but changes in their expression may cause mitochondrial DNA instability and could lead to mitochondrial disease.

## Genes encoding proteins involved in nucleotide metabolism

Balance in free nucleotide concentrations is very important for proper DNA replication. It is particularly important in mitochondria, because there are no de novo nucleotide biosynthesis pathways. Mitochondria rely mainly on salvage pathways localized partially in mitochondria and partially in the cytoplasm. Imbalance in free nucleotide concentrations leads to disturbances in mtDNA replication and in consequence to mtDNA copy number decrease or to the appearance of multiple deletions.

There are two deoxyribonucleoside kinases expressed in mitochondria phosphorylating purine and pyrimidine deoxyribonucleosides. Thymidine kinase-2 (TK2) phosphorylates deoxythymidine, deoxycytidine, and deoxyuridine, while deoxyribonucleoside kinase (dGK) phosphorylates deoxyguanosine and deoxyadenosine (Saada et al. 2001). The *RRM2B* gene encodes a protein participating in catalytic conversion of ribonucleoside diphosphates (NDP) to deoxyribonucleoside diphosphates (dNDP) – basic elements for DNA synthesis (Pontarin et al. 2012).

### TK2

The *TK2* gene (16q21, 42,410 bp, 12 exons) encodes a mitochondrial matrix enzyme – thymidine kinase 2 (TK2). TK2 is an enzyme essential for mtDNA maintenance, catalyzes the rate-determining step of the pyrimidine salvage pathway (Tyynismaa et al. 2012) and generates (by phosphorylation) thymidine monophosphate (TMP), cytidine monophosphate (CMP) and deoxyuridine from deoxypyrimidine nucleosides (Cámara et al. 2015; Saada et al. 2001).

Mutations in the *TK2* gene result in a decrease of enzyme activity which impairs recycling of mtDNA nucleotides and finally causes progressive muscle weakness (myopathy) and mitochondrial DNA depletion syndrome 2 (myopathic type, MIM 609560) (Cámara et al. 2015; Saada et al. 2001; Wang et al. 2003).

Approximately 30 pathogenic mutations in the *TK2* gene have been described (ClinVar) with a hot spot in exon 5 (Manusco et al. 2003).

For example, Cámara et al. (2015) observed that compound mutations in the *TK2* gene (p.T108 M and p.K202del) were present in DNA isolated from muscle biopsies from patients with myopathy. They also observed a dramatic decrease of mtDNA copy number in cells. Structural analysis of the enzyme showed that missense mutations were linked with binding affinities of dTMP and dCTP (Cámara et al. 2015). Mutation p.T108 M was also described by Behin et al. (2012) and Paradas et al. (2013) and was associated with a 30% depletion of mtDNA and deletion of 45% of mtDNA molecules when compared to controls.

### DGUOK

Deoxyguanosine kinase (dGK), another matrix enzyme, encoded by the *DGUOK* gene (2p13, 32,136 bp, 8 exons) provides phosphorylated purines necessary for mtDNA synthesis (Jullig and Eriksson 2000; Ronchi et al. 2012). Mutations in this gene lead to mitochondrial DNA depletion syndrome 3 (hepatocerebral type) (MIM 251880).

In silico analysis suggested that the most frequent mutations in the gene affect the structure of dGK. Biochemical analysis of the activity of dGK isolated from skeletal muscles from myopathic patients showed that mutations may impair the enzyme function (Ronchi et al. 2012).

### RRM2B

*RRM2B* gene (8q22.3, 34,618 bp, 9 exons) encodes ribonucleotide reductase regulatory TP53 inducible subunit M2B (p53R2), a part of ribonucleotide reductase. This cytoplasmic enzyme is responsible for conversion of ribonucleoside diphosphates to deoxyribonucleoside diphosphates and is an element of the de novo nucleotide biosynthesis pathway. Ribonucleotide reductase is composed of large R1 and small R2 subunits. There are two types of R2 subunits in the cell. One is present during the S phase of the cell cycle only, the second one, encoded by *RRM2B*, provides the basal level of deoxyribonucleoside diphosphates. mtDNA replication, generally independent of cell cycle, takes place not only during the S phase, p53R2 protein is crucial for mtDNA synthesis.

Mutations in *RRM2B* lead to mtDNA depletion (Pontarin et al. 2012).

Diseases associated with mutations in the *RRM2B* gene include mitochondrial DNA depletion syndrome 8A (encephalomyopathic type with renal tubulopathy) (MIM 612075), mitochondrial DNA depletion syndrome 8B (MNGIE type) (MIM 612075), and progressive external ophthalmoplegia with mitochondrial DNA deletions, autosomal dominant 5 (MIM 613077).

### TYMP

Thymidine phosphorylase (TP) is another protein important for nucleotide biosynthesis encoded by the *TYMP* gene (22q13.33, 4334 bp, 10 exons). This cytosolic enzyme of the salvage pathway catalyzes the cleavage of thymidine into thymine and 2-deoxy-α-D-ribose-1-phosphate and plays an important role in catabolic processes (Javaida et al. 2016). TP is also considered a promoter of tumor growth and metastasis. Overexpression prevents apoptosis and induces angiogenesis and is associated with tumor aggressiveness and poor prognosis (Bronckaers et al. 2009).

Mutations in *TYMP* lead to accumulation of nucleosides and an imbalance in the mitochondrial nucleotide pool and results in MNGIE type autosomal recessive mitochondrial DNA depletion syndrome 1 (MIM 603041) (Table 1).

**Table 1:** Nuclear genes involved in mtDNA instability

Gene	Localization	MIM number	Disease	Inheritance	Phenotype	Age of onset
*MNF2*	1p36.22	609,260	Charcot-Marie-Tooth disease, axonal, type 2A2A (CMT2A2A)	AD	CMT disease is a group of progressive neurologic disorders characterized by peripheral neuropathy and optic atrophy. Damage of the peripheral nerves results in loss of sensation (touch, pain, heat, and sound) and muscle weakness in the feet, legs, and hands	infancy
	1p36.23	617,087	Charcot-Marie-Tooth disease, axonal, type 2A2B (CMT2A2B)	AR		infancy
	1p36.24	601,152	Hereditary motor and sensory neuropathy VIA (CMT6A)	AD		childhood or adulthood
*DGUOK*	2p13.1	251,880	Mitochondrial DNA depletion syndrome 3 (hepatocerebral type)	AR	Genetic disorder characterized by multisystemic neurological abnormalities including muscle weakness, PEO, liver failure and lactic acidosis	infancy
	2p13.2	617,068	Portal hypertension, noncirrhotic	AR	Disorder, relatively benign, is characterized by onset of high blood pressure in the hepatic portal system associated with hepatosplenomegaly.	childhood or adulthood
	2p13.3	617,070	Progressive external ophthalmoplegia with mitochondrial DNA deletions, autosomal recessive 4	AR	Disorder characterized by adult onset of eye muscle weakness and proximal limb muscle weakness	adulthood
*MPV17*	2p23.3	256,810	Mitochondrial DNA depletion syndrome 6 (hepatocerebral type)	AR	Infantile onset disorder which affects liver and muscles.	infancy
*RNASEH1*	2p25.3	616,479	Progressive external ophthalmoplegia with mitochondrial DNA deletions, autosomal recessive 2	AR	Disorder characterized by adult onset of PEO, proximal limb muscle weakness and symptoms of spinocerebellar ataxia	adulthood
*MFF*	2q36.3	617,086	Encephalopathy due to defective mitochondrial and peroxisomal fission 2	AR	Encephalopathy, including delayed psychomotor development, hypotonia and muscle weakness	childhood
*OPA1*	3q29	616,896	Mitochondrial DNA depletion syndrome 14 (encephalocardiomyopathic type)	0	Encephalocardiomyopathy.	infancy
	3q30	210,000	Behr syndrome	AR	Optic atrophy associated with neurological manifestations including myoclonic epilepsy, progressive spastic paraplegia, dysarthria, extra-pyramidal tract signs, ataxia, urinary incontinence, mental retardation, posterior column sensory loss or muscle contractures (predominant in the lower limbs)	early childhood
	3q31	165,500	Optic atrophy 1	AD	Optic atrophy with onset of visual impairment in early childhood	early childhood
	3q32	125,250	Optic atrophy plus syndrome	AD	Optic atrophy with PEO and ataxia (and wide range of intermediate phenotypes)	childhood
*PRIMPOL*	4q35.1	615,420	Myopia 22, autosomal dominant	AD	Eye abnormality where light focuses in front of the retina and causes nearsightedness.	early childhood
*SLC25A4*	4q35.1	617,184	Mitochondrial DNA depletion syndrome 12A (cardiomyopathic type) AD	AD	Disorders characterized by cardiomyopathy or hypertrophic cardiomyopathy and muscle weakness	infancy
	4q35.2	615,418	Mitochondrial DNA depletion syndrome 12B (cardiomyopathic type) AR	AR		childhood
	4q35.3	609,283	Progressive external ophthalmoplegia with mitochondrial DNA deletions, autosomal dominant 2	AD	Disorder characterized by weakness of the external eye muscles, limb muscle weakness and exercise intolerance	adulthood
*RRM2B*	8q22.3	612,075	Mitochondrial DNA depletion syndrome 8A (encephalomyopathic type with renal tubulopathy)	AR	Mitochondrial neurogastrointestinal encephalopathy (MNGIE). Disease affects the digestive and nervous system	infancy
	8q22.4	612,075	Mitochondrial DNA depletion syndrome 8B (MNGIE type)			
	8q22.5	613,077	Progressive external ophthalmoplegia with mitochondrial DNA deletions, autosomal dominant 5	AD	Autosomal dominant progressive external ophthalmoplegia (adPEO)	adulthood
*TFAM*	10q21.1	617,156	Mitochondrial DNA depletion syndrome 15 (hepatocerebral type)	AR	First symptoms occur at or soon after birth including hypoglycemia, hyperbilirubinemia, jaundice etc.	infancy
*DNA2*	10q21.3	615,807	Seckel syndrome 8	AR	Also known as bird-headed dwarfism, disorder characterized by growth and mental retardation	infancy
	10q21.4	615,156	Progressive external ophthalmoplegia with mitochondrial DNA deletions, autosomal dominant 6	AD	Adult onset PEO with limb-girdle muscle weakness predominantly affecting the lower limb	childhood or adulthood
*TWNK*	10q24.31	271,245	Mitochondrial DNA depletion syndrome 7 (hepatocerebral type)	AR	Neurodegenerative disease characterized by hypotonia, ataxia, ophthalmoplegia, hearing loss, seizures, and sensory axonal neuropathy	childhood or adulthood
	10q24.32	616,138	Perrault syndrome 5	AR	Neurological disorder with a characteristic feature of hearing loss caused by abnormalities in the inner ear	childhood
	10q24.33	609,286	Progressive external ophthalmoplegia with mitochondrial DNA deletions, autosomal dominant 3	AD	Clinical features of this disease include adult onset of weakness of the external eye muscles and exercise intolerance	adulthood
*DNM1L*	12p11.21	614,388	Encephalopahty, lethal, due to defective mitochondrial peroxisomal fission 1	AD, AR	Encephalopathy with hypotonia and delayed psychomotor development	childhood
*POLG*	15q26.1	203,700	Mitochondrial DNA depletion syndrome 4A (Alpers type)	AR	Alpers syndrome is the clinical triad of psychomotor retardation, intractable epilepsy, and liver failure in infants and young children	infancy to young children
	15q26.2	613,662	Mitochondrial DNA depletion syndrome 4B (MNGIE type)	AR	Progressive multisystem disorder clinically characterized by chronic gastrointestinal dysmotility, PEO, axonal sensory ataxic neuropathy and muscle weakness	childhood or adulthood
	15q26.3	607,459	Mitochondrial recessive ataxia syndrome (includes SANDO and SCAE)	AR	SANDO is characterized by sensory ataxic neuropathy, dysarthria, and ophthalmoparesis.	adulthood
	15q26.4	157,640	Progressive external ophthalmoplegia, autosomal dominant 1	AD	PEO and muscle weakness, may include hearing loss, ataxia and parkinsonism	adulthood
	15q26.5	258,450	Progressive external ophthalmoplegia, autosomal recessive 1	AR		adulthood
*TK2*	16q21	617,069	Progressive external ophthalmoplegia with mitochondrial DNA deletions, autosomal recessive 3	AR	Adult-onset progressive external ophthalmoplegia, sometimes with progressive proximal muscle weakness	adulthood
	16q22	609,560	Mitochondrial DNA depletion syndrome 2 (myopathic type)	AR	Childhood onset of muscle weakness and a slowly progressive myopathy	infancy or childchood
*SPG7*	16q24.3	607,259	Spastic paraplegia 7	AD, AR	Disease characterized by spasticity of limbs sometimes with additional neurologic features	adulthood
*POLG2*	17q23.3	610,131	Progressive external ophthalmoplegia with mitochondrial DNA deletions, autosomal dominant 4	AD	PEO and variably affected skeletal muscle, the nervous system, the liver, and the gastrointestinal tract	infancy to adulthood
*MGME1*	20p11.23	615,084	Mitochondrial DNA depletion syndrome 11	AR	Disorder characterized by PEO, muscle weakness and atrophy	childhood or adulthood
*TYMP*	22q13.33	603,041	Mitochondrial DNA depletion syndrome 1 (MNGIE type)	AR	Progressive multisystem disorder clinically characterized by PEO, gastrointestinal dysmotility, cachexia, diffuse leukoencephalopathy and peripheral neuropathy	adulthood

AR – autosomal recessive, AD – autosomal dominant

### SLC25A4

The *SLC25A4* gene (4q35.1, 7144 bp, 4 exons) encoding a heart muscle specific isoform of a solute carrier family 25 (mitochondrial carrier, adenine nucleotide translocator) member 4 also known as *ANT1* was the first gene in which mutations responsible for mtDNA instability were described (Kaukonen et al. 2000). The gene product (monomer protein) forms a pore at the mitochondrial inner membrane. ANT1 predominates in post-mitotic tissues such as muscles and heart (Pebay-Peyroula et al. 2003; Ahmed et al. 2015). ANT1 regulates ATP and ADP transport — it transfers ADP from the cytoplasm to the mitochondrial matrix and ATP from the matrix to the cytoplasm (Neckelmann et al. 1987; Kawamata et al. 2011). The exact mechanism of mtDNA destabilization by the *SLC25A4* mutations is not known. Definitely, the ADP/ATP balance is important for maintenance of the membrane potential. It is also postulated that ATP and ADP concentration may influence dATP quantity and also affect proper DNA/RNA hybrid formation during replication.

Most of the described *SLC25A4* mutations cause misfolding of the protein and affect intermembrane exchange of molecules leading to inhibition of cell growth. Moreover, the changed ANT1 protein interacts with other membrane proteins and affects their function (Liu et al. 2015).

Mitochondrial DNA instability was shown in *SLC25A4* knockout mice (Krishnan et al. 2008).

Mutations in *SLC25A4* can be inherited in an autosomal dominant or recessive manner. Dominant mutations leading to progressive external ophthalmoplegia with mitochondrial DNA deletions (PEO) (MIM 609283) seem to show a dominant-negative effect on the molecular level, while the phenotype caused by recessive ones (mitochondrial DNA depletion syndrome 12 (cardiomyopathic type)) (MIM 615418) is more similar to the one obtained for knockout mice.

## Genes encoding proteins involved in mitochondrial fusion, fission, and mobility

Mitochondria are no longer considered as static bean-shaped structures. They move, fuse, and divide and form a network interconnected with the endoplasmic reticulum. Undisturbed fusion, fission, and movement are especially important in neurons, where mitochondria have to travel along axons and dendrites to act in the proper time and place. Fusion and fission are energy dependent so they rely on effective respiration and at the same time quality control of mitochondria is based on fusion and fission. Defects in fusion, as proven in detail in mice, lead to loss of mtDNA copy number, multiple mtDNA deletions, and increase the point mutation rate (Chen et al. 2010). Genes involved in mitochondrial fusion and fission most frequently mentioned in the context of human disease include *OPA* (OPA1, mitochondrial dynamin like GTPase), *MFN1* (mitofusin 1), and *MFN2* (mitofusin 2) encoding proteins involved in fusion, *FIS1* (mitochondrial fission 1 protein), *DNM1L* (dynamin 1-like protein, Drp1), and *MFF* (mitochondrial fission factor) important for proper mitochondrial division (MacVicar and Langer 2016; Losón et al. 2013).

### OPA1

The *OPA1* (3q29, 104,668 bp, 32 exons) gene encodes mitochondrial dynamin like GTPase. OPA1 plays an important role in mtDNA maintenance, mutations in the *OPA1* gene lead to mtDNA multiple deletions (Hudson et al. 2007). OPA1 protein localizes to the inner mitochondrial membrane where it is involved in cristae formation and proper fusion of the inner membrane. One of the OPA1 isoforms localizes in the nucleoid and seems to be involved in mtDNA replication (Yu-Wai-Man et al. 2010; Elachouri et al. 2011).

More than 200 mutations in the *OPA1* gene have been found of both autosomal dominant and autosomal recessive character. They are associated with autosomal dominant optic atrophy 1 (MIM 165500), optic atrophy plus syndrome (MIM 125250) and autosomal recessive Behr syndrome (MIM 210000). In one consanguineous family, mitochondrial DNA depletion syndrome 14 (encephalocardiomyopathic type) (MIM 616896) due to a homozygous *OPA1* mutation has been described.

### MFN1

The *MFN1* gene (3q26.33, 47,253 bp, 17 exons) encodes a transmembrane GTPase localized in the mitochondrial outer membrane. MNF1 (mitofusin 1) forms homomultimers and heteromultimers with MFN2 (mitofusin 2) and together they are responsible for outer mitochondrial membrane fusion. There are no human diseases attributed to *MFN1* mutations.

### MFN2

Similar to *MFN1*, the *MFN2* gene (1p36.22, 33,335 bp, 19 exons) encodes a transmembrane GTPase localized in the mitochondrial outer membrane and shares high homology with MFN1. Two transcriptional forms are known. Mitofusin 2 was described as a protein enabling close contact of mitochondria with the endoplasmic reticulum. Recently the opposite was found: depletion of MFN2 results in a closer contact with ER (Filadi et al. 2015).

Mutations in *MFN2* are the main cause of autosomal dominant Charcot-Marie-Tooth disease, axonal, type 2A2A (MIM 609260) but also autosomal dominant hereditary motor and sensory neuropathy VIA (MIM 601152) and autosomal recessive Charcot-Marie-Tooth disease, axonal, type 2A2B (MIM 617087). mtDNA depletion and multiple deletions were observed in muscles and fibroblasts from patients with AD Charcot-Marie-Tooth disease caused by *MFN2* mutations (Vielhaber et al. 2013).

### DNM1L

The *DNM1L* (12p11.21, 66,451 bp, 21 exons) encodes another member of the GTPase family: dynamin-1-like protein which regulates mitochondrial function and plays a crucial role in the division, fusion, and fission of mitochondria. DNM1L forms an oligomeric ring at the division spot that narrows and splits the mitochondrial tubule (Fahrner et al. 2016). *DNM1L* is critically important in human (and general mammalian) development, its absence causes abnormality in embryonic development. Nonsense mutations disturb central nerve system development. Yoon et al. (2016) have shown that if mutations were present in the *DNML* gene, giant mitochondria with an abnormal shape were present in neurons in human (with compound heterozygous mutations in *DNM1L* gene) and knock-out mice. Mutations in the DNM1L gene cause encephalopathy which is lethal due to defective mitochondrial and peroxisomal fission (type 1) (MIM 614388, autosomal dominant).

### MFF

The *MFF* gene encodes the mitochondrial fission factor (2q36.3, 32,686 bp, 13 exons), which is an outer membrane protein required for localization of DNM1L and division of mitochondria. MFF protein together with FIS1 are responsible for recruitment of DNM1L to the division site (Friedman et al. 2011).

Mutations in *MFF* lead to a similar phenotype to DNM1L mutations (encephalopathy, lethal, due to defective mitochondrial peroxisomal fission 2, autosomal recessive).

### FIS1

FIS1 – tetratricopeptide repeat domain-containing protein 11 encoded by the *FIS1* gene (7q22.1, 5479 bp, 5 exons) acts independently of MFF. No diseases caused by FIS1 mutations have been described.

## Miscellaneous

Besides the above-mentioned genes encoding proteins involved in the processes with more or less well described influence on mtDNA stability, there are multiple other genes not involved in any of these processes in which mutations lead to mtDNA deletions or depletion. Here we mention only a few of them with the highest impact on human health.

### MPV17

Although *MPV17* (2p23.3, 13,611 bp, 9 exons) mutations were described as a cause of autosomal recessive mitochondrial depletion syndrome ten years ago (Spinazzola et al. 2006), the function of the protein encoded by this gene was not known. Recently (Antonenkov et al. 2015), this inner membrane protein was shown to function as a non-selective channel under a strict control of factors reflecting the energetic state of mitochondria such as membrane potential or redox state.

### SPG7

The *SPG7* gene (16q24.3, 66,852 bp, 22 exons) encodes paraplegin which is a component of the mitochondrial AAA protease. Spastic paraplegia 7 takes part in many cellular functions like ribosome assembly regulation, membrane trafficking, protein folding, intracellular motility, organelle biogenesis, and proteolysis. Mutations in *SPG7* historically have been attributed to spastic paraplegia 7, autosomal recessive (MIM 607259) but recently were found to be an important factor in mitochondrial diseases (Sánchez-Ferrero et al. 2013; Pfeffer et al. 2014; Gorman et al. 2015). Mutations in that gene lead to chronic progressive external ophthalmoplegia due to disordered mitochondrial DNA maintenance. In the North East England population prevalence of mutations in the *SPG7* gene is greater than in TWNK, *OPA1*, and *POLG* genes (Pfeffer et al. 2015; Gorman et al. 2015).

As we mentioned at the beginning of this review, mitochondrial diseases are very difficult to diagnose due to complex genotype–phenotype relationships, also called a blended phenotype. This means that mutations in one gene can lead to different clinical phenotypes and mutations in different genes can lead to the same signs and symptoms (Wortmann et al. 2015).

## Phenotype

**Fig. 2:**
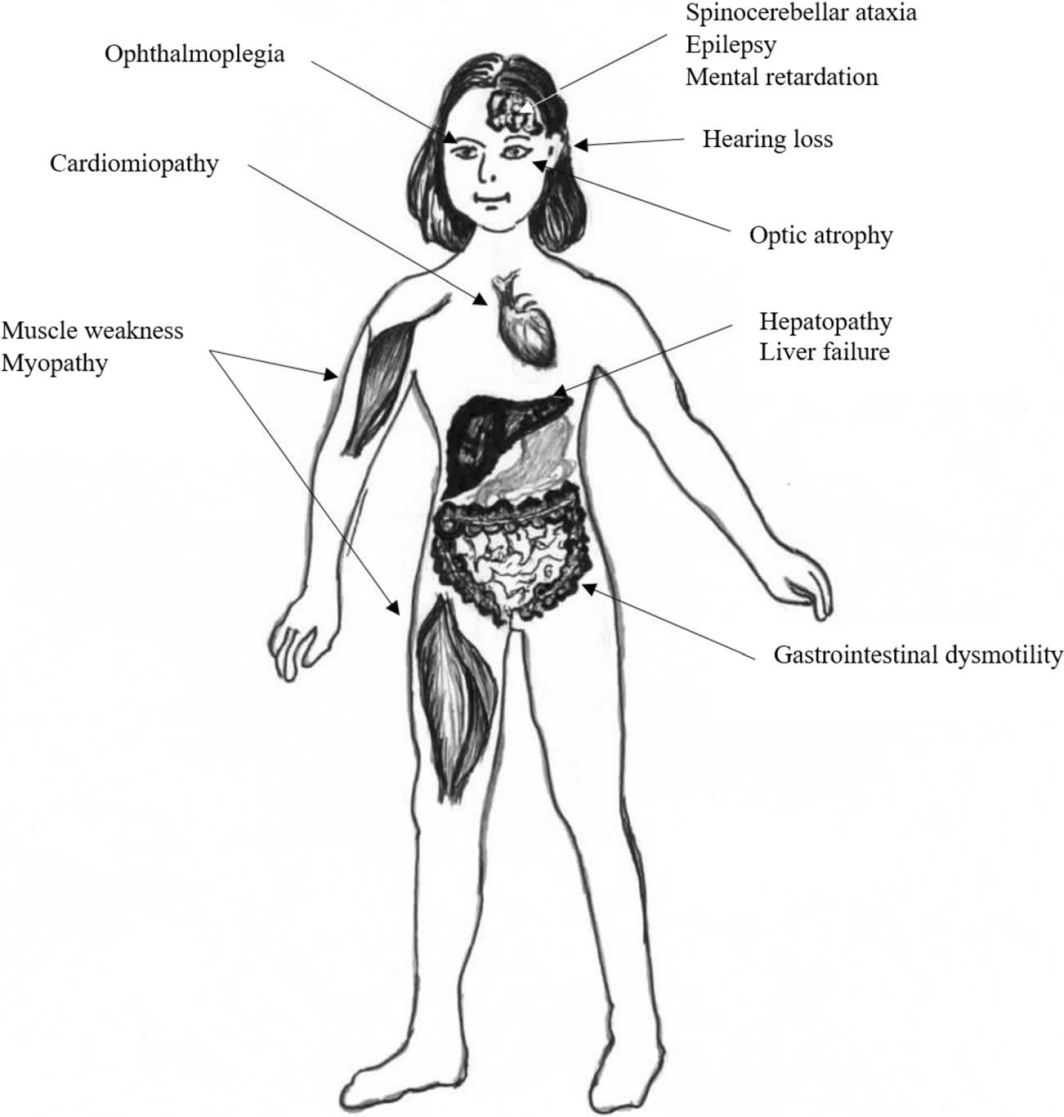
The main symptoms of the diseases caused by mitochondrial DNA instability

Mitochondrial diseases affect each individual differently. Although mitochondrial disease primarily affects children, adult onset is becoming more common (Fig. 2).

## Summary

Mitochondrial diseases are a heterogeneous group of diseases. Age of onset is very different, from infants to the fifth decade of life. Symptoms involve multiple tissues and most of them may be progressive. Genetic background of this group of diseases in most cases is still unknown. The list of genes involved in mitochondrial DNA maintenance is long and still incomplete.

Although therapeutic options are still limited, effective diagnosis on the clinical and molecular level opens the way to proper treatment. In some cases, targeted therapy is possible such as nucleotide supplementation in the case of patients with *TK2* mutations.
